# Nanocellulose-Based Patches Loaded with Hyaluronic Acid and Diclofenac towards Aphthous Stomatitis Treatment

**DOI:** 10.3390/nano10040628

**Published:** 2020-03-28

**Authors:** João P. F. Carvalho, Ana C. Q. Silva, Verónica Bastos, Helena Oliveira, Ricardo J. B. Pinto, Armando J. D. Silvestre, Carla Vilela, Carmen S. R. Freire

**Affiliations:** 1 CICECO—Aveiro Institute of Materials, Department of Chemistry, University of Aveiro, 3810-193 Aveiro, Portugal; joao.pedro.carvalho@ua.pt (J.P.F.C.); ana.cristina.silva@ua.pt (A.C.Q.S.); r.pinto@ua.pt (R.J.B.P.); armsil@ua.pt (A.J.D.S.); 2 Department of Biology & CESAM, University of Aveiro, 3810-193 Aveiro, Portugal; veronicabastos@ua.pt (V.B.); holiveira@ua.pt (H.O.)

**Keywords:** bacterial nanocellulose, hyaluronic acid, diclofenac, nanostructured patches, drug delivery, aphthous stomatitis

## Abstract

Nanostructured patches composed of bacterial nanocellulose (BNC), hyaluronic acid (HA) and diclofenac (DCF) were developed, envisioning the treatment of aphthous stomatitis. Freestanding patches were prepared via diffusion of aqueous solutions of HA and DCF, with different concentrations of DCF, into the wet BNC three-dimensional porous network. The resultant dual polysaccharides-based patches with a nanostructured morphology present thermal stability up to 200 °C, as well as good dynamic mechanical properties, with a storage modulus higher than 1.0 GPa. In addition, the patches are non-cytotoxic to human keratinocytes (HaCaT cells), with a cell viability of almost 100% after 24 h. The in vitro release profile of DCF from the patches was evaluated in simulated saliva, and the data refer to a diffusion- and swelling-controlled drug-release mechanism. The attained results hint at the possibility of using these dual polysaccharides-based oral mucosal patches to target aphthous stomatitis.

## 1. Introduction

Recurrent aphthous stomatitis (RAS) is the most common form of ulceration of the oral mucosa, affecting from 5% to 66% of the world’s population [1]. RAS, also known as aphthae or canker sores, is characterized by the presence of round or oval ulcers with circumscribed margins and an erythematous halo [2]. These ulcers usually last merely for a few days before healing spontaneously, but they might frequently reoccur. Nevertheless, these generally small wounds can be extremely uncomfortable, causing stinging pain while speaking, eating or drinking [3,4]. The current treatment of RAS aims to mitigate the symptoms, especially the tingling or burning pain, and inflammation caused by the ulcers [5]. Multiple formulations for topical use have been described, employing a wide array of substances, including anti-inflammatories, analgesics, antimicrobials and healing-promoting agents, in the form of gel-like formulations, sprays and mouthwashes [2,5].

For instance, the effect of hyaluronic acid (HA), viz. a linear polysaccharide of D-glucuronic acid and *N*-acetyl-D-glucosamine [6,7], on the treatment of RAS is known [2,4,8]. The healing potential of this biocompatible, biodegradable, non-immunogenic and mucoadhesive polysaccharide in RAS is credited to the enhancement of tissue regeneration and the formation of a physical barrier protecting the wound [2,8,9]. Still, the extreme discomfort derived from aphthae is mainly caused by the stinging pain, and HA does not possess quick analgesic potential [10]. The concomitant use of HA with drugs for rapid pain-relief and anti-inflammatory effects, namely non-steroid anti-inflammatory drugs (NSAIDs), probably constitutes the best option for the fast relief and healing in a RAS case [2]. Diclofenac (DCF) is an NSAID frequently used for its analgesic effect in short-term clinical situations (like musculoskeletal complaints or after dental work) and in long-term treatment of rheumatoid arthritis [11,12], but also to reduce the RAS pain [2]. As an illustrative example, Saxen et al. [10] combined HA with DCF to obtain a gel-like formulation for the treatment of aphthous ulcers with positive results in pain-reduction in these cases. Nonetheless, these gel, cream or paste formulations have some drawbacks, since they wash away from the target area, need to be applied several times a day in order to form a physical protective layer over the RAS, and originate side-effects in the long-term use [2].

Herein, membrane- or film-like formulations would be a more adequate option to develop buccal carrier devices. Even so, these kinds of formulations are still an underrated domain, with very few alternatives successfully developed [13,14,15,16]. Among the natural substrates that can be used to engineer membranes or films, bacterial nanocellulose (BNC), viz. an exopolysaccharide produced by some non-pathogenic bacteria, namely the acetic acid bacteria of the genus *Komagataeibacter* (formerly classified as *Gluconacetobacter*) [17,18], is gaining increasing attention in the biomedical realm [19,20,21], particularly as a wound-dressing material [19,22,23]. Hence, the current study was inspired not only by the biocompatibility, high water-retention capacity, nanostructured porous network and good in vivo skin compatibility of BNC [20,24], but also by the fact that this exopolysaccharide can be directly produced in the form of membranes or films with customizable size and shape, and can house an array of active molecules (e.g., lidocaine [25,26], diclofenac [27,28], amoxicillin [29] and levofloxacin [30]) and macromolecules (e.g., poly([2-(methacryloyloxy)ethyl]trimethylammonium chloride) [31] and vitamin B-based ionic liquids [32]) that confer new functionalities to the ensuing materials. Although (i) HA has already been added to the culture media during BNC biosynthesis, to obtain BNC/HA membranes with no specific application [33], and (ii) the combination between BNC and DCF has already been studied for transdermal delivery [27], the coalition of BNC with HA and DCF has not yet been studied, at least to the best of our knowledge, for the potential treatment of aphthous stomatitis.

In this perspective, the present work portrays the production of BNC-based patches containing both HA and DCF, aiming for the simultaneous mitigation of pain and stimulation of healing of the aphthous ulcers in RAS. The freestanding membrane patches were fabricated via simple diffusion of HA and DCF aqueous solutions into the wet BNC three-dimensional porous network. An elaborate characterization of the structure, morphology, thermal stability, dynamic mechanical properties, and moisture- and water-uptake capacity of the patches is exposed, as well as the in vitro cytotoxicity towards human HaCaT keratinocyte cells and drug-release profile in simulated salivary fluid.

## 2. Materials and Methods

### 2.1. Chemicals, Materials and Cells

Diclofenac sodium salt (DCF, ≥98.5%), potassium sulphate (K_2_SO_4_, ≥99.0%), 3-(4,5-dimethylthiazol-2-yl)-2,5-diphenyltetrazolium bromide (MTT, 98%) and dimethyl sulfoxide (DMSO, ≥99.9%) were obtained from Sigma-Aldrich (Sintra, Portugal). Hyaluronic acid sodium salt (HA, MW 403.31 kDa, >95%) was acquired from Molekula (München, Germany). Agarose basic was purchased from AppliChem (Darmstadt, Germany). Dulbecco’s Modified Eagle’s Medium (DMEM), fetal bovine serum (FBS), phosphate buffer solution (PBS, pH 7.4), L-glutamine, penicillin/streptomycin and fungizone were obtained from Gibco^®^ (Life Technologies, Carlsbad, CA, USA). Ultrapure water (Type 1, 18.2 MΩ·cm at 25 °C) was purified by a Simplicity^®^ Water Purification System (Merck, Darmstadt, Germany). Other chemicals and solvents were of laboratory grade.

Bacterial nanocellulose (BNC) in the form of wet membranes (ca. 99% water) that were around 7 cm in diameter was produced in our laboratory, using the *Gluconacetobacter sacchari* strain, maintained under conventional culture conditions [34]. The HaCaT cells, a line of nontumorigenic immortalized human keratinocytes, were obtained from Cell Lines Services (Eppelheim, Germany).

### 2.2. Preparation of Nanocellulose-Based Patches

Wet BNC membranes were weighed (ca. 200 mg on a dry basis), and nearly 60% of their water content was removed with laboratory-grade absorbent paper. The drained membranes were then soaked in 12 mL of aqueous solutions containing HA (0.2% w/v) and DCF (0.5 and 1.0% w/v), as enumerated in Table 1, and were left for 24 h at room temperature (RT), to fully incorporate the respective solution. For comparison purposes, patches containing solely HA or DCF with the same concentrations were also prepared (Table 1). After the total absorption of the solutions (viz. 100% entrapment efficiency), the resulting patches were left to dry in a ventilated oven (Thermo Fisher Scientific, USA) at 40 °C for 16 h. All patches were prepared in triplicates and kept in a desiccator until further use.

### 2.3. Characterization Methods

The thickness of the free-standing patches was measured at several random sites, using a Mitutoyo coolant-proof digimatic micrometer MDC-25PX (Mitutoyo Corporation, Tokyo, Japan).

Fourier transform infrared–attenuated total reflection (FTIR–ATR) spectra were collected with a PerkinElmer FT-IR System Spectrum BX spectrophotometer (PerkinElmer Inc., Waltham, MA, USA) equipped with a single horizontal Golden Gate ATR cell, over the range of 600–4000 cm^−1^, at a resolution of 4 cm^−1^, over 32 scans.

Scanning electron microscopy (SEM) images of the surface and cross-section (fractured in liquid nitrogen) of the samples were obtained by a HR-FESEM SU-70 Hitachi microscope (Hitachi High-Technologies Corporation, Tokyo, Japan) operating at 4 kV. The samples were placed on a aluminum plate and previously coated with a carbon film.

Thermogravimetric analysis (TGA) was carried out with a SETSYS Setaram TGA analyzer (SETARAM Instrumentation, Lyon, France) equipped with a platinum cell. The samples were heated from RT to 800 °C, at a constant rate of 10 °C min^−1^ under inert (N_2_) atmosphere.

Dynamic mechanical analysis (DMA) curves of rectangular membrane pieces with 3 × 0.5 cm^2^ were obtained on a Tritec 2000 DMA (Triton Technologies, London, UK) operating in tension mode (single strain) at 1 Hz and with 0.005 mm displacement. The temperature was swept from −50 to 150 °C, with a constant heating rate of 2 °C min^−1^.

### 2.4. Moisture- and Water-Uptake Capacity

The moisture-uptake capacity was assessed by placing the dry patch specimens (2 × 2 cm^2^) in a conditioned cabinet, at about 98% relative humidity (RH, saturated potassium sulphate aqueous solution, 97.59 ± 0.53% [35]), at RT for 24 and 48 h. After removing the specimens from the chamber, the weight (*W_w_*) was measured, and the moisture-uptake was calculated according to the following equation:

$$Moisture\;uptake\;\left(\%\right)=\left(W_{w}-W_{0}\right)\times W_{0}^{-1}\times 100$$

where *W_0_* is the initial weight of the dry patch.

The water-uptake capacity was evaluated by putting the dry patch specimens (2 × 2 cm^2^) in contact with an agarose hydrogel (1.4% w/w, as physical skin model [36]) at RT for 24 h. After removing the membrane patches from the surface of the agarose hydrogel, the wet samples’ weight (*W_w_*) was measured. The water-uptake was calculated by the following equation:

$$Water\;uptake\;\left(\%\right)=\left(W_{w}-W_{0}\right)\times W_{0}^{-1}\times 100$$

where *W_0_* is the initial weight of the dry patch.

Three replicas of each sample were simultaneously tested for both assays.

### 2.5. In Vitro Cytotoxicity Assay

The cytotoxicity of the patches was evaluated in a human keratinocytes cell line (HaCaT cells) by using the MTT assay [37]. Briefly, cells were grown in complete DMEM supplemented with 10% FBS, 2 mM L-glutamine, 10,000 U mL^-1^ penicillin/streptomycin and 250 µg mL^−1^ fungizone, at 37 °C, in 5% CO_2_ humidified atmosphere. Cells were daily observed under an inverted-phase-contrast Eclipse TS100 microscope (Nikon, Tokyo, Japan). The tests were performed for BNC, BNC/HA_0.2 and BNC/HA/DCF_1.0, and, as a negative control, HaCaT cells were treated identically, as described for the samples, but exposed only to DMEM medium. Two independent assays, with 6 replicates each, were carried out.

Patch samples of 1 × 1 cm² were prepared, sterilized by ultraviolet (UV) radiation and then incubated with 2 mL of complete DMEM medium at 37 °C, with 5% CO_2_, for 24 h, to prepare the sample extract. HaCaT cells were seeded in a 96-well plate, at 6500 cells/well, and exposed for 24 h to the extracts of BNC, BNC/HA_0.2 and BNC/HA/DCF_1.0, obtained from the incubated samples. At the end of the incubation time, 50 μL of MTT (1 g L^−1^) was added to each well and incubated for 4 h, at 37 °C, in 5% CO_2_ humidified atmosphere. After that, culture medium with MTT was removed and replaced by 150 μL of DMSO, and the plate was placed in an orbital shaker for 2 h, in the dark, to completely dissolve the formazan crystals. The absorbance of the samples was measured with a BioTek Synergy HT plate reader (Synergy HT Multi-Mode, BioTeK, Winooski, VT, USA) at 570 nm, with blank corrections. The cell viability was calculated with respect to the control cells:

$$Cell\;viability\;\left(\%\right)=\left[\left(Abs_{sample}-Abs_{DMSO}\right)/\left(Abs_{control}-Abs_{DMSO}\right)\right]\times 100$$

where $Abs_{sample}$ is the absorbance of the sample, $Abs_{DMSO}$ is the absorbance of the DMSO solvent and $Abs_{control}$ is the absorbance of the control.

### 2.6. In Vitro Diclofenac Release Assay

The drug-release study was performed in 50 mL of simulated salivary fluid (SS2, pH 7.4 [38]), at 37 °C, under magnetic stirring, at 130 rpm, and with patch samples with dimensions of 2 × 2 cm^2^. Aliquots of 2 mL were collected at predetermined time points, and the collected medium was always replaced with the same volume of fresh medium (preheated at 37 °C). Three replicas were simultaneously performed for each sample.

The release of DCF into the media was evaluated by determining drug-concentration through UV-Vis spectroscopy (Thermo Scientific Evolution UV-Vis 600, Thermo Fisher Scientific, Waltham MA, USA) at 276 nm. The cumulative release concentration was calculated by using the following formula:

$$C_{cumulative}=C_{n}+\left[\left(2\times C_{n-1}\right)/50\right]$$

where *C_n_* and *C_n−1_* are the concentrations of DCF in solution at times n and n − 1. A calibration curve $\left(y=0.0165x+0.0604;\;R^{2}=0.99983\right)$ was obtained at 276 nm for DCF, in the range of 1–60 μg mL^–1^.

### 2.7. Statistical Analysis

Analysis of variance (ANOVA) and Tukey’s test (OriginPro, version 9.0.0, OriginLab Corporation, Northampton, MA, USA) were used to determine the statistical significance established at *p* < 0.05.

## 3. Results and Discussion

Two nanostructured patches composed of BNC, HA and DCF were prepared by impregnation of the wet BNC three-dimensional porous membrane with aqueous solutions of HA and DCF. HA was selected due to its ability to enhance the healing of several types of damaged tissues, including oral wounds [39], whereas DCF was carefully chosen because of its local analgesic effect in short-term clinical situations [11]. HA was used in a concentration of 0.2% (w/v) [2,40], while for DCF, two concentrations were chosen, namely 0.5% and 1.0% (w/v) [27], all based on equivalent commercial formulations. For comparison purposes, patches containing solely HA (0.2% w/v) and DCF (0.5 and 1.0% w/v) were also prepared (Table 1).

**Figure 1:**
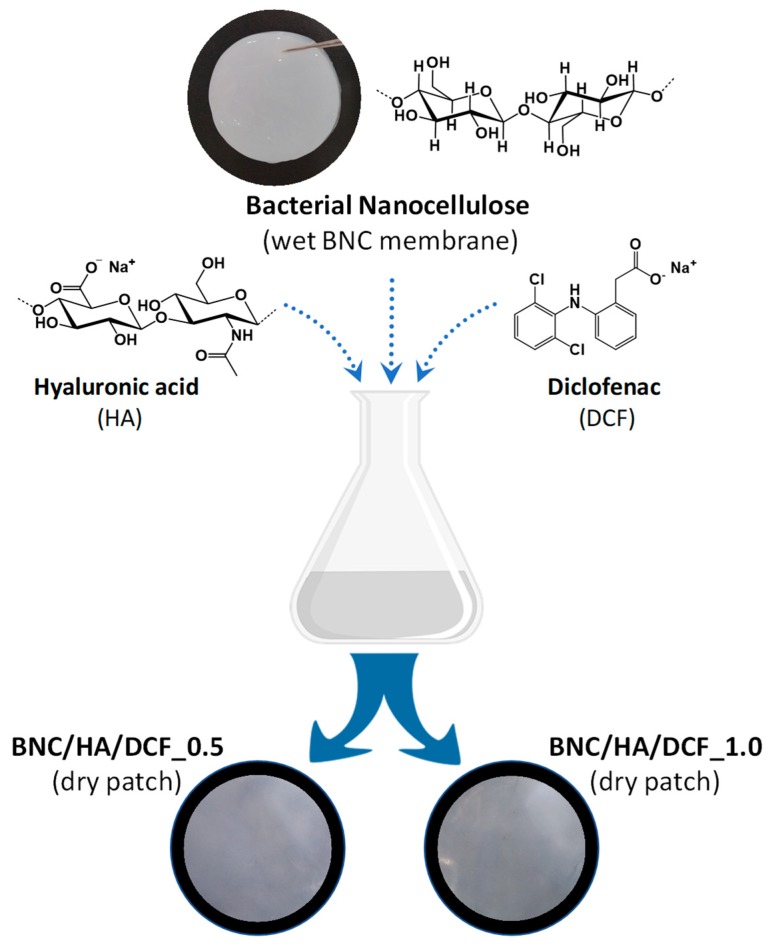
Scheme of the preparation of the bacterial nanocellulose/hyaluronic acid/diclofenac (BNC/HA/DCF) membrane patches.

The two patches, viz. BNC/HA/DCF_0.5 and BNC/HA/DCF_1.0, are composed of 0.62 mg of HA and 1.56 mg of DCF per cm^2^ of patch, and 0.62 mg of HA and 3.12 mg of DCF per cm^2^ of patch, respectively. Furthermore, the patches are pearly, uniform and homogeneous (Figure 1), and they present thickness values of 79 ± 12 μm and 83 ± 8 μm, respectively (Table 1). All patches were characterized in the matter of structure (FTIR–ATR spectroscopy), morphology (SEM), thermal stability (TGA), dynamic mechanical properties (DMA), and moisture- and water-uptake capacity. In addition, the in vitro cytotoxicity and drug release assays were also evaluated.

### 3.1. Structure and Morphology

**Figure 2:**
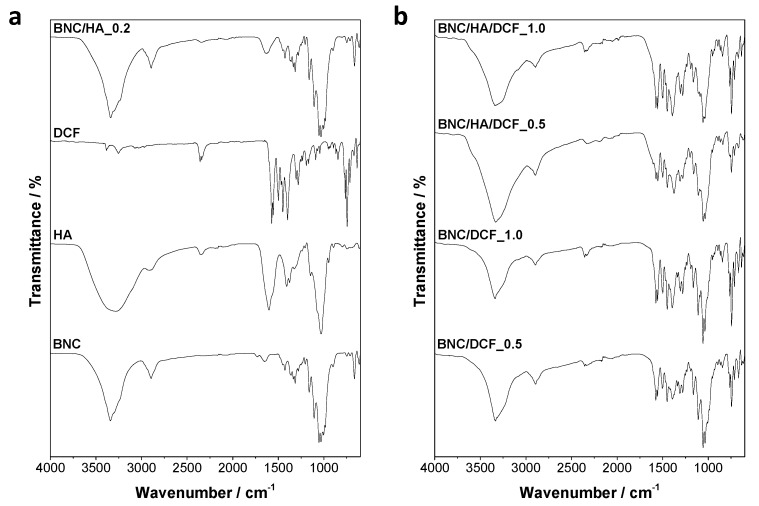
Fourier transform infrared–attenuated total reflection (FTIR–ATR) spectra of (**a**) BNC, HA, DCF, BNC/HA_0.2, and (**b**) BNC/DCF_0.5, BNC/DCF_1.0, BNC/HA/DCF_0.5 and BNC/HA/DCF_1.0.

The FTIR–ATR spectra of the two BNC/HA/DCF patches and the corresponding precursors are portrayed in Figure 2. The cellulosic substrate (BNC, Figure 2a) presents absorption bands at 3341 cm^−1^, allocated to the O–H stretching vibration of the primary and secondary hydroxyl groups; 2898 cm^−1^ assigned to the stretching vibration of the C–H bonds; 1315 cm^−1^ ascribed to the O–H in plane bending vibration of the primary and secondary hydroxy groups; 1160 cm^−1^ attributed to the C–O–C asymmetric stretching vibration of the glycosidic bonds, and 1031 cm^−1^ assigned to the C–O stretching vibration [41]. The HA spectrum (Figure 2a) is characterized by the absorption peaks at 3296 cm^−1^ (O–H and N–H stretching), 2910 cm^−1^ (CH symmetric and CH_2_ asymmetric stretching), 1608 cm^−1^ (N–H bending of amide II and COO^–^ asymmetric stretching), 1376 cm^−1^ (COO^−^ symmetric vibration) and 1032 cm^−1^ (C–O stretching) [42,43]. The spectrum of DCF (Figure 2a) reveals its distinctive absorption bands at 3382 cm^−1^ (N–H stretching), 1572 cm^−1^ (COO^–^ asymmetrical vibration), 1350–1250 cm^−1^ (C–N stretching) and 745–730 cm^−1^ (C–H out-of-plane, di- and tri-substituted rings) [28,44].

The spectra of the BNC/HA/DCF patches (Figure 2b) exhibit the absorption bands characteristic of the three precursors, namely BNC, HA and DCF. Although the content of HA (0.2% w/v) is quite small in all patches, the strong vibrational band of HA at 1630 cm^−1^ (N–H bending of amide II and COO^–^ asymmetric stretching) is clearly visible in the BNC/HA patch but less evident in the BNC/HA/DCF patches due to the overlap with the vibrations of DCF. In fact, the strong vibrational bands of DCF at 1574 cm^−1^ (COO^–^ asymmetrical vibration) and 744 cm^−1^ (C–H out-of-plane, di-and tri-substituted rings) are clearly perceptible in the spectra of both BNC/DCF and BNC/HA/DCF patches (Figure 2b). Furthermore, the intensity of the absorption bands allocated to DCF increases with the increasing content of DCF from 0.5% to 1.0% (w/v, Figure 2b). Hence, the inclusion of HA and DCF into the BNC three-dimensional porous network was effectively accomplished.

**Figure 3:**
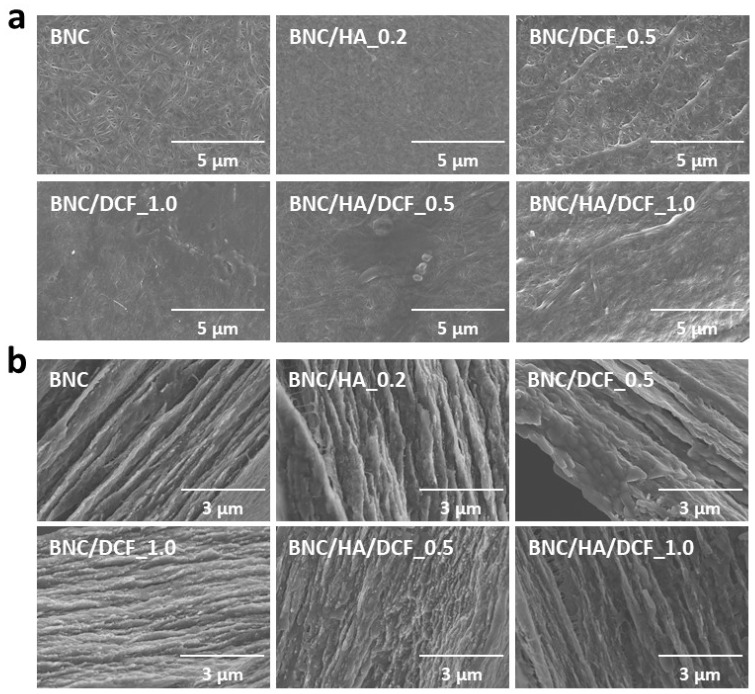
Scanning electron microscopy (SEM) micrographs of the (**a**) surface (×10.0 k magnification) and (**b**) cross-section (×15.0 k magnification) of BNC, BNC/HA_0.2, BNC/DCF_0.5, BNC/DCF_1.0, BNC/HA/DCF_0.5 and BNC/HA/DCF_1.0.

The morphology of all membranes was studied by SEM with the surface and cross-sectional micrographs compiled in Figure 3. The characteristic three-dimensional nanofibrillar structure of BNC [45,46] is clearly visible on both the surface and cross-sectional micrographs of the pure membrane. No obvious difference was observed in the micrographs of the patch containing only HA (BNC/HA_0.2), given the small content of anionic polysaccharide incorporated into the BNC porous structure (Figure 3a,b). In contrast, the morphology of the patches containing DCF, namely BNC/DCF_0.5 and BNC/DCF_1.0, is different from the pure BNC membrane, since the fibrillar and lamellar structure is being slightly covered with the increasing content of DCF. The same was observed for the patches composed of HA and DCF, i.e., BNC/HA/DCF_0.5 and BNN/HA/DCF_1.0 (Figure 3a,b), given that the BNC porous structure was packed with both HA and DCF components. This aspect is in line with facts stated for other BNC-based materials containing, for instance, vitamin-B-based ionic liquids and fucoidan [32,46].

### 3.2. Thermal and Mechanical Properties

**Figure 4:**
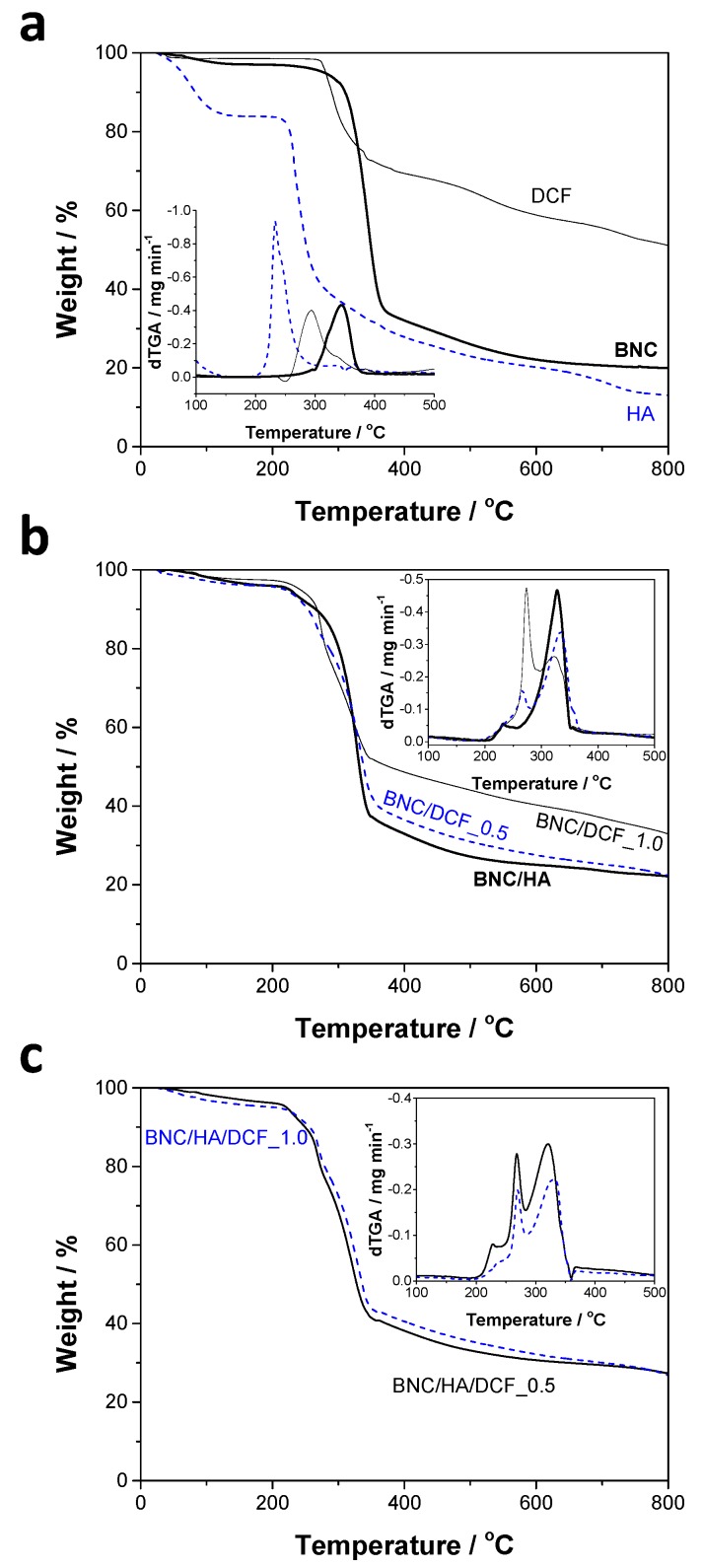
Thermogravimetric curves of (**a**) BNC, HA, DCF, (**b**) BNC/HA_0.2, BNC/DCF_0.5, BNC/DCF_1.0, (**c**) BNC/HA/DCF_0.5 and BNC/HA/DCF_1.0 under nitrogen atmosphere. The inset curves correspond to the derivative.

The thermal stability of the patches and their precursors was assessed by TGA under inert atmosphere, viz. nitrogen. According to the data provided in Figure 4a, BNC exhibited the typical profile of a nanocellulose substrate with a single weight-loss degradation step with initial and maximum decomposition temperature of 290 and 344 °C, respectively, and a final residue of 20% at 800 °C [47]. In the case of HA, the TGA profile is also characterized by a single weight-loss step with initial and maximum decomposition temperatures of 200 and 233 °C, respectively, but it exhibits a substantial dehydration step at ca. 100 °C, with a weight loss of about 15 wt.% (Figure 4a). This feature confirms the highly hygroscopic nature of this anionic polysaccharide [48] and the data obtained here agree with those reported in the literature [49]. DCF also presents a single-step weight-loss curve with initial and maximum decomposition temperatures of 260 and 292 °C, respectively, with a final residue of ca. 50% at 800 °C (Figure 4a). For the BNC/HA and BNC/DCF patches, the thermograms present a two-step weight-loss profile, with each step corresponding to the primary components, as depicted in Figure 4b.

Regarding the BNC/HA/DCF patches (Figure 4c), both display a three-step weight-loss degradation profile, apart from the dehydration below 100 °C (ca. 2–3 wt.% weight loss). The first step, at about 228 °C, for BNC/HA/DCF_0.5 and 236 °C for BNC/HA/DCF_1.0, is allocated to the HA degradation; the second step, at around 268 °C, for BNC/HA/DCF_0.5 and 270 °C for BNC/HA/DCF_1.0, is assigned to DCF degradation; and the third step, at ca. 321 °C, for BNC/HA/DCF_0.5 and 328 °C for BNC/HA/DCF_1.0, corresponds to the degradation of BNC. Both patches are thermally stable up to 200 °C and have a final residue of ca. 27%. Although the thermal stability of the BNC/HA/DCF patches is lower when compared with the pristine BNC membrane, which is a common trend in other BNC-based nanomaterials [47], it is fair to state that these patches can be safely submitted to sterilization processes (e.g., autoclaving at ca. 150 °C) compulsory in biomedical applications.

**Figure 5:**
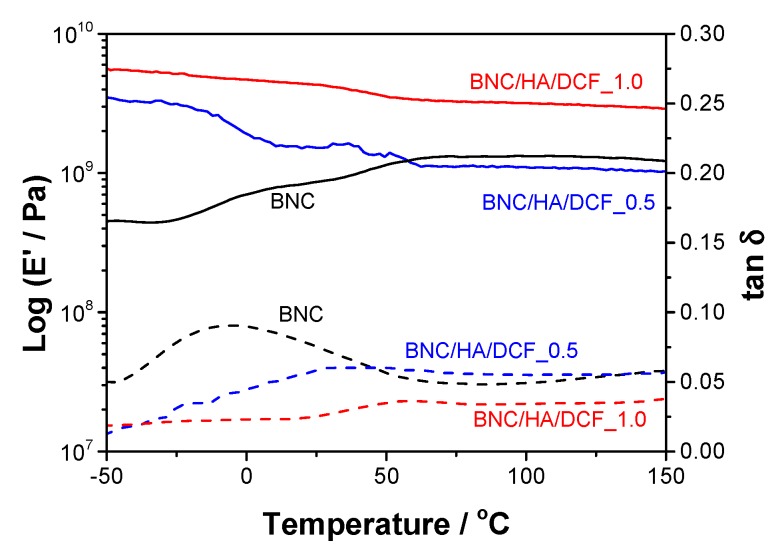
Storage modulus (*E*’, — solid line) and loss factor (tan *δ*, --- dashed line) of BNC, BNC/HA/DCF_0.5 and BNC/HA/DCF_1.0.

The dynamic mechanical properties of the patches were assessed by DMA from –50 to 150 °C, and the tensile storage modulus (*E*’) and loss-factor (tan *δ*) results are outlined in Figure 5. The data obtained for the pure BNC membrane shows an increase in the *E*’ values, from 0.45 GPa at –50 °C to 1.2 GPa at 150 °C, as well as a step between ca. –31 and 15 °C that parallels with the loss-factor peak in the range of –46 to 53 °C. This tan *δ* peak might be correlated with the plasticizing effect of water [50] by taking into account that the pure BNC membrane contains roughly 2% of water, as verified by TGA (Figure 4a). Concerning the BNC/HA/DCF membrane patches, the *E*’ of BNC/HA/DCF_0.5 drops from 3.5 GPa at –50 °C to 1.0 GPa at 150 °C, whereas for the BNC/HA/DCF_1.0 the *E*’ values decrease from 5.6 GPa at –50 °C to 2.9 GPa at 150 °C. Despite the drop with temperature increase, both patches exhibit good dynamic mechanical performance, particularly in the range of 35–36 °C, i.e., the median temperature of the human oral cavity [51], with *E*’ values of about 1.6 GPa for BNC/HA/DCF_0.5 and 4.0 GPa for BNC/HA/DCF_1.0. Interestingly, these patches might also withstand the temperature fluctuation during oral function (e.g., drinking and eating), with extreme values of around 6–7 °C and 54–58 °C [51].

### 3.3. Moisture- and Water-Uptake Capacity

**Figure 6:**
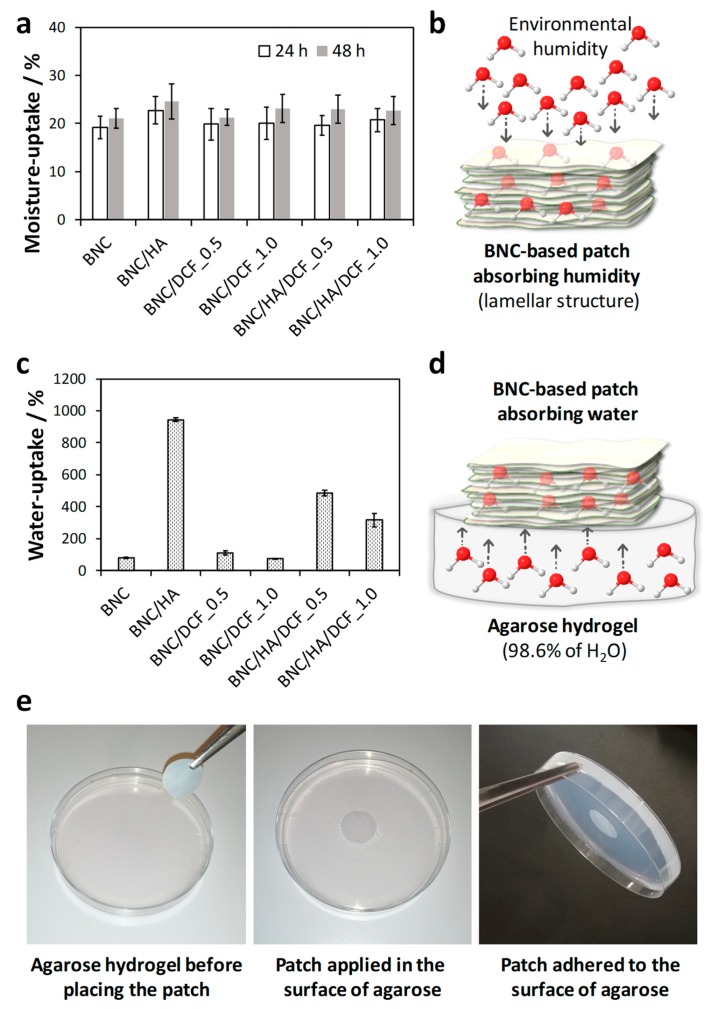
(**a**) Moisture-uptake capacity of BNC, BNC/HA_0.2, BNC/DCF_0.5, BNC/DCF_1.0, BNC/HA/DCF_0.5 and BNC/HA/DCF_1.0 after 24 and 48 h; (**b**) scheme showing the absorption of environmental humidity; (**c**) water-uptake capacity of BNC, BNC/HA_0.2, BNC/DCF_0.5, BNC/DCF_1.0, BNC/HA/DCF_0.5 and BNC/HA/DCF_1.0 after 24 h; (**d**) scheme evidencing the absorption of water from the agarose hydrogel; and (**e**) photographs of the BNC/HA/DCF_1.0 patch in contact with agarose hydrogel (1.4% w/w) to simulate the patch adherence to the oral mucosa.

The moisture-uptake capacity of the patches was determined to predict their interaction with environmental humidity. Therefore, the patches were placed in a chamber with controlled humidity, i.e., 98% RH [35], at room temperature. As anticipated, the hydrophilic BNC absorbs environmental humidity with values of 19 ± 2% after 24 h and 21 ± 2% after 48 h (Figure 6a). These results concur with data reported in previous studies [46,52].

The inclusion of 0.2% (w/v) of HA into the BNC membrane originated a small increment in the moisture-uptake capacity to 23 ± 3% after 24 h and 25 ± 4% after 48 h, because HA is a hygroscopic anionic polysaccharide [48], as in fact shown in the TGA analysis (Figure 4a). On the contrary, the incorporation of only DCF and HA/DCF into the BNC porous membrane did not have a significant effect on the moisture-uptake, as evidenced in Figure 6a and confirmed by the fact that the means’ difference is not significant. Still, these patches are able to absorb environmental humidity (Figure 6b), which is a relevant property for application in the oral mucosa.

Therefore, given the ability of these BNC-based patches to absorb moisture, the subsequent phase was to test their ability to absorb water from a skin model that might mimic the presence of mucus and saliva. Herein, agarose hydrogel was selected as a versatile and easy-to-produce physical skin model, with a density similar to that of human skin [36]. All patches adhered to the surface of the agarose hydrogel composed of 98.6% of water, as illustrated in Figure 6e for BNC/HA/DCF_1.0, and simultaneously absorbed water from the agarose hydrogel (Figure 6c,d). The BNC/HA patch is the one with the highest water-uptake capacity (943 ± 11%), followed by BNC/HA/DCF_0.5 with 484 ± 19%, BNC/HA/DCF_1.0 with 315 ± 43%, BNC/DCF_0.5 with 110 ± 11% and BNC/DCF_1.0 with 72 ± 2%. Furthermore, and given the fact that BNC does not disintegrate when exposed to aqueous media, the membrane patches retained their mechanical integrity after water absorption during 24 h, which agrees with the dynamic mechanical properties (Figure 5). These results clearly show the ability of the BNC/HA/DCF patches to interact with mucus and saliva when placed in contact with an aphtha in the oral mucosa and adhere to it (Figure 6e).

### 3.4. In Vitro Cytotoxicity

**Figure 7:**
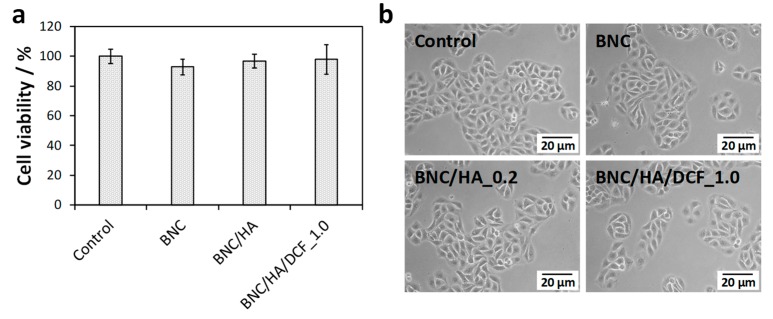
(**a**) Cell viability (the difference of the means is not significant at the 0.05 level) and (**b**) optical micrographs of HaCaT cells after 24 h of exposure to negative control, BNC, BNC/HA_0.2 and BNC/HA/DCF_1.0 patches.

The cytotoxicity of the patch with the higher content of DCF, viz. BNC/HA/DCF_1.0, as well as of BNC and BNC/HA_0.2, was evaluated in human HaCaT keratinocyte cells through the indirect MTT assay [37]. This cell line was adopted because it has been applied in many studies as a model for epidermal cells [28,31,53], including the oral mucosa [54]. According to the data provided in Figure 7a, the HaCaT cells’ metabolic activity after 24 h of exposure to BNC membrane (93±5% cell viability) is similar to that of the negative control (100% cell viability). Hence, the BNC membrane is non-cytotoxic to HaCaT cells, which is consistent with the results obtained in literature for this cell line [28,31], but also for other cell lines, e.g., RAW 264.7 cells [55] and adipose-derived stem cells (ADSCs) [56].

When the BNC membrane is loaded solely with HA, the cell viability is almost unaffected with a value of 97 ± 5% after 24 h. In fact, this was anticipated considering that HA is non-cytotoxic to HaCaT cells [57] and other cell lines [58], and thus is employed in several pharmaceutical formulations for both cosmetic and pharmaceutical purposes. In the case of the patch with the higher content of both HA and DCF (i.e., BNC/HA/DCF_1.0), it is also considered a non-cytotoxic material, since the cell viability was 98 ± 10% after 24 h (Figure 7a), way above the 70% threshold of cell viability [59]. The optical micrographs of the HaCaT cells (Figure 7b) clearly corroborate the cell viability values by showing that neither the cell morphology nor the cell count is altered when compared with the control after 24 h of cell incubation with all patches. These results give an indication of the potential in vivo behavior of these oral mucosal patches, which are safe and compatible for biomedical applications.

### 3.5. In Vitro Drug Release

**Figure 8:**
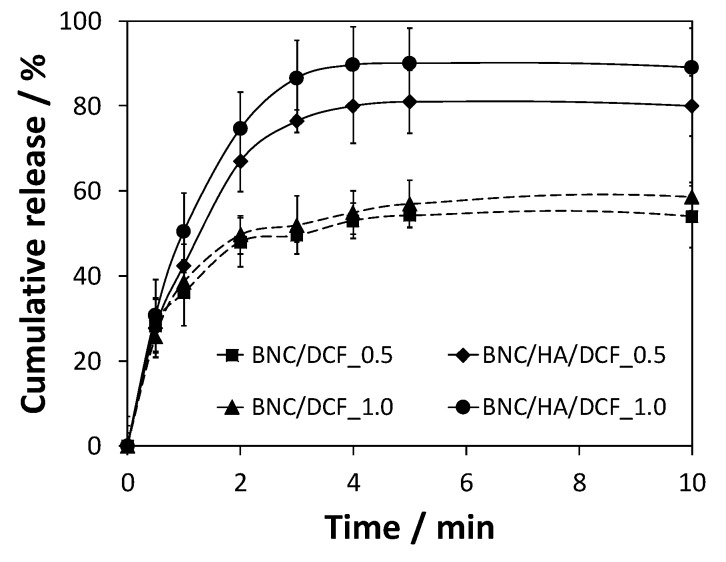
DCF cumulative release profile of the BNC/DCF_0.5, BNC/DCF_1.0, BNC/HA/DCF_0.5 and BNC/HA/DCF_1.0 patches.

The in vitro release of diclofenac from the two BNC/HA/DCF membrane patches was quantified in simulated salivary fluid [38], at 37 °C, and compared with the in vitro release of DCF from patches composed solely of BNC and DCF. Overall, all patches revealed a standard release profile, with a burst, followed by a plateau, where the DCF release reaches the highest value, as represented in Figure 8. Although none of the patches achieved a total release of DCF (i.e., 100% cumulative release), the BNC/HA/DCF_1.0 reached the maximum cumulative release of 90% after 4 min. On the other hand, the BNC/HA/DCF_0.5 only reached a maximum cumulative release of 81% after 5 min. When compared with the other two systems, namely BNC/DCF_0.5 and BNC/DCF_1.0, the BNC/HA/DCF patches exhibit a much faster release rate with BNC/HA/DCF_1.0, exhibiting the fastest one. This is certainly associated with the high water-uptake of the BNC/HA/DCF patches, as previously discussed (Figure 6c). Actually, these release values are similar to those reported for BNC membranes loaded with DCF and plasticized with glycerol [27,60]. Still, this rapid release profile is adequate for the therapeutic effect intended in this study. In fact, a rapid local analgesic and anti-inflammatory action can be achieved by ensuring a fast-transcutaneous penetration, which is only possible if an appropriate amount of DCF is released from the patch.

The DCF release profiles of the BNC/HA/DCF patches (Figure 8) can be fitted to the Korsmeyer–Peppas kinetic model [61,62]: *M_t_*/*M_∞_* = *kt^n^*, where *M_t_* is the amount of DCF released at time *t*, *M_∞_* is the amount of DCF released at infinite time, *k* is the kinetic constant and *n* is the diffusion constant, indicating the release mechanism [61,63]. Based on this model, only the values of *M_t_*/*M_∞_* < 60% are fitted; hence, a release exponent (*n*) of 0.61 (regression coefficient: *R*^2^ = 0.9999) was achieved for BNC/HA/DCF_0.5 and 0.71 (*R*^2^ = 0.9999) for BNC/HA/DCF_1.0. These fitting parameters are representative of an anomalous or non-Fickian transport (0.5 < *n* < 1.0), which translates into a diffusion- and swelling-controlled drug-release mechanism [61,62,63].

The data gathered in the present study show a portfolio of adjustable properties, namely thermal stability, dynamic mechanical properties, water-uptake capacity, non-cytotoxicity and DCF release profile, that depend on the content of the primary components of the BNC/HA/DCF patches. The blend between (i) an exopolysaccharide directly produced in the form of membranes with a nanostructured porous network (i.e., BNC), (ii) a mucoadhesive polysaccharide with tissue regeneration ability (i.e., HA) and (iii) a non-steroid anti-inflammatory and analgesic drug (i.e., DCF) created patches with potential for the simultaneous mitigation of pain and stimulation of healing of the aphthous ulcers.

## 4. Conclusions

Nanostructured patches composed of two polysaccharides, viz. BNC and HA, and a non-steroid anti-inflammatory and analgesic drug, viz. DCF, were developed for the potential treatment of aphthous stomatitis. Two freestanding patches were prepared via simple and easy-to-use diffusion of aqueous solutions of HA and DCF into the wet BNC three-dimensional porous network. These nanostructured patches have thermal stability up to 200 °C and adequate dynamic mechanical properties with a minimum storage modulus of 1.0 GPa. In addition, the patches are non-cytotoxic to human keratinocytes (HaCaT cells), with a cell viability of almost 100% after 24 h. The in vitro release profile of DCF from the patches was tested in simulated salivary fluid, and the data point to a diffusion- and swelling-controlled drug-release mechanism. All these figures validate the potentiality of these dual polysaccharides-based oral mucosal patches to target aphthous stomatitis.

## Figures and Tables

**Table 1:** List of patches with the respective compositions and thickness values.

Membrane Patch	HA/% ^a^	DCF/% ^a^	Thickness/μm
BNC	–	–	39 ± 8
BNC/HA_0.2	0.2	–	45 ± 9
BNC/DCF_0.5	–	0.5	51 ± 4
BNC/DCF_1.0	–	1.0	79 ± 15
BNC/HA/DCF_0.5	0.2	0.5	79 ± 12
BNC/HA/DCF_1.0	0.2	1.0	83 ± 8

^a^ w/v: mass of HA or DCF per volume of aqueous solution.
